# *EGFR*突变晚期非小细胞肺癌患者后线接受免疫治疗的疗效分析

**DOI:** 10.3779/j.issn.1009-3419.2021.104.06

**Published:** 2021-05-20

**Authors:** 丽 马, 娜 秦, 新勇 张, 羽华 吴, 浩洋 李, 孟军 俞, 子臣 刘, 敬慧 王

**Affiliations:** 1 101149 北京, 首都医科大学附属北京胸科医院, 北京市结核病胸部肿瘤研究所肿瘤内科 Department of Medical Oncology, Beijing Chest Hospital, Capital Medical University/Beijing Tuberculosis and Thoracic Tumor Research Institute, Beijing 101149, China; 2 101149 北京, 首都医科大学附属北京胸科医院, 北京市结核病胸部肿瘤研究所病理科 Department of Pathology, Beijing Chest Hospital, Capital Medical University/Beijing Tuberculosis and Thoracic Tumor Research Institute, Beijing 101149, China; 3 101149 北京, 首都医科大学附属北京胸科医院, 北京市结核病胸部肿瘤研究所肿瘤研究中心 Cancer Research Center, Beijing Chest Hospital, Capital Medical University/Beijing Tuberculosis and Thoracic Tumor Research Institute, Beijing 101149, China

**Keywords:** 肺肿瘤, 表皮生长因子受体, 基因突变, 免疫治疗, Lung neoplasms, Epidermal growth factor receptor, Gene mutation, Immunotherapy

## Abstract

**背景与目的:**

免疫检查点抑制剂单药治疗在驱动基因阳性的晚期非小细胞肺癌（non-small cell lung cancer, NSCLC）患者中疗效甚微。研究表明，部分驱动基因阳性患者靶向治疗耐药后对免疫联合治疗仍有效。国内研究甚少。本研究旨在分析人表皮生长因子（epidermal growth factor receptor, *EGFR*）敏感突变NSCLC患者后线接受免疫治疗的疗效，评价真实世界免疫联合化疗在*EGFR*突变晚期患者后线治疗中的价值。

**方法:**

收集2018年6月-2020年11月在首都医科大学附属北京胸科医院确诊的*EGFR*突变的初治晚期肺腺癌患者共27例，均在靶向治疗进展后接受了程序性死亡受体1（programmed cell death protein 1, PD-1）检查点抑制剂联合化疗以及抗血管生成药物治疗。

**结果:**

27例晚期NSCLC患者中，未合并T790M突变的有19例（70.4%），合并T790M点突变的有8例（29.6%）。总客观缓解率为40.7%。*Kaplan-Meier*生存分析显示，不同*EGFR*突变类型之间接受含PD-1单抗治疗的无进展生存期（progression-free survival, PFS）均无统计学差异（*χ*^2^=4.15, *P*=0.23）。未合并T790M突变的患者PFS较合并T790M突变的患者显著延长（9.2个月*vs* 3.3个月，*χ*^2^=2.81，*P*=0.041），两者总生存时间未见统计学差异（12.2个月*vs* 7.3个月，*χ*^2^=3.22，*P*=0.062）。未合并T790M的客观缓解率明显优于合并T790M的患者（52.63% *vs* 12.5%, *P*=0.045）。

**结论:**

*EGFR*突变患者人群能从后线免疫联合治疗中获益，但合并T790M突变的患者后线接受免疫联合治疗疗效差。因此，这部分患者的后续治疗和全程化管理需要探索更优的治疗策略来提高获益。

## Abstract (en)

**Background and objective:**

Immune checkpoint inhibitor monotherapy is reported to have little effect in advanced non-small cell lung cancer (NSCLC) patients with driver oncogenes. However, recent studies have shown that some patients with driver genes are still benefit from combination immunotherapy after tyrosine kinase inhibitors (TKIs) drug resistance. The purpose of this study was to analyze the efficacy of posterior line immunotherapy in NSCLC patients with epidermal growth factor (*EGFR*) sensitive mutation, and to evaluate the value of immunotherapy in posterior line therapy in patients with advanced *EGFR* mutation.

**Methods:**

A total of 27 patients with *EGFR* mutation diagnosed in Beijing Chest Hospital, Capital Medical University from June 2018 to November 2020 were collected. After the progress of targeted therapy, they had received programmed cell death protein 1 (PD-1) checkpoint inhibitor combined with chemotherapy and anti-angiogenic drug therapy.

**Results:**

Of the 27 advanced NSCLC patients, 19 cases (70.4%) did not have T790M mutation. There were 8 cases (29.6%) with T790M point mutation. The total objective response rate (ORR) was 40.7%. *Kaplan-Meier* survival analysis showed that there was no statistically significant difference among different *EGFR* mutations (*χ*^2^=4.15, *P*=0.230). But progression-free survival (PFS) was significantly longer in patients without T790M mutation than in patients with T790M mutation (9.2 mon *vs* 3.3 mon, *χ*^2^=2.808, *P*=0.041), and the same trend was observed in patients with overall survival treated with the PD-1 inhibitor (12.2 mon *vs* 7.3 mon, *χ*^2^=3.22, *P*=0.062). ORR of patients without T790M was significantly better than that with T790M (52.63% *vs* 12.5%, *P*=0.045).

**Conclusion:**

Patients with *EGFR* mutation can benefit from later-line combined immunotherapy. The patients with T790M mutation in the population of *EGFR* mutation had the worst effect of immunotherapy in the later line. Therefore, the follow-up treatment and whole-course management of these patients need to explore better treatment strategies to improve the benefit.

人表皮生长因子受体（epidermal growth factor receptor, EGFR）酪氨酸激酶拮抗剂（tyrosine kinase inhibitor, TKI）是治疗携带*EGFR*敏感突变晚期非小细胞肺癌（non-small cell lung cancer, NSCLC）患者的一线标准治疗，但大部分患者会在12个月内发生耐药，耐药后的治疗方法有限^[1, 2]^。自2018年以来，随着免疫治疗在不同瘤种的广泛应用，研究者们试图寄希望于将免疫治疗应用到*EGFR*突变的人群中^[3, 4]^，但前期研究^[5]^表明，*EGFR*突变的人群由于生物学特性不同，驱动型癌基因导致免疫抑制激活的通路较多，免疫微环境存在较多抑制信号。*EGFR*突变人群一度被认为是“免疫豁免”人群。一些临床试验和*meta*分析^[4, 6]^也提示单药免疫治疗*EGFR*突变人群的获益有限，疗效均劣于EGFR野生型患者。然而，IMpower150研究^[7]^为了探索不同免疫联合方案在*EGFR*突变人群中的价值，设计了*EGFR*突变的亚组，研究结果表明，程序性死亡配体1（programmed death-ligand 1, PD-L1）抑制剂联合化疗和抗血管生成治疗的总生存时间（overall survival, OS）较化疗联合抗血管生成治疗的患者显著延长（29.4个月*vs* 18.1个月，HR=0.6，95%CI：0.31-1.41）。这一国际多中心研究颠覆了人们对“*EGFR*突变免疫豁免”的理论，为免疫治疗在*EGFR*突变人群中的应用增加了信心，也为EGFR-TKI耐药的患者带来新的治疗选择。因此，本研究通过评估人源化的程序性死亡受体1（programmed death protein 1, PD-1）单抗联合化疗以及抗血管生成治疗*EGFR*突变的NSCLC患者的疗效，为*EGFR*突变患者靶向治疗耐药后的治疗策略和全程管理提供临床经验。

## 1 对象与方法

### 1.1 研究对象

收集自2018年6月-2020年11月的晚期肺腺癌病例。纳入标准：①病理确诊为肺腺癌；②组织学或细胞学基因检测曾有*EGFR*敏感突变；③曾接受EGFR-TKI药物口服治疗；④美国东部肿瘤协作组（Eastern Cooperative Oncology Group, ECOG）评分0分-2分；⑤均为EGFR-TKI靶向治疗进展的患者，临床分期Ⅲb期-Ⅳ期[根据第8版肿瘤原发灶-淋巴结-转移（tumor-node-metastasis, TNM）分期]；其中，一代、二代药物进展后，若有T790M突变的患者接受奥西替尼治疗进展后；⑥有完整的临床病理信息，包括吸烟、性别、年龄和临床分期等；⑦TKI进展后接受了免疫治疗（PD-1抑制剂）联合化疗和抗血管生成治疗。排除标准：①年龄 < 18岁；②已怀孕女性；③合并其他恶性肿瘤病史；④有间质性肺炎等免疫治疗禁忌证。共纳入27例晚期肺腺癌患者。

### 1.2 基因检测方法

#### 1.2.1 突变扩增系统（amplification refractory mutation system, ARMS）技术检测患者肿瘤组织和血液中*EGFR*基因突变类型

每例患者在入组时均已明确*EGFR*基因突变类型。由我院病理科提供石蜡切片标本，QIAamp DNA试剂盒定量提取石蜡切片肿瘤组织的DNA^[9]^。通过ARMS-PCR技术检测肿瘤组织中*EGFR*基因突变类型，厦门艾德公司ADx-ARMS试剂盒检测29种突变类型，试剂盒设有内部和外部质控样本，阳性对照和阴性对照。通过Super-ARMS技术检测血液标本中*EGFR*基因突变情况^[10]^。

#### 1.2.2 检测方法

肿瘤组织或细胞学标本用4%的甲醛溶液浸泡，经过包埋固定后制成石蜡切片。苏木精-伊红（Hematoxylin-Eosin, HE）染色，在显微镜下病理学专家进行细胞类型确诊，保证至少80%以上的肿瘤细胞成分。QIAamp DNA试剂盒定量提取石蜡切片肿瘤组织的DNA。收集外周血至一管10 mL EDTA-K2抗凝管中，在2 h内处理血标本。全血1, 600 rpm离心10 min。收集上清液后以16, 000 rpm 4 ℃离心10 min。此次收集的上清液为血浆，分装保存至-80 ℃。利用QIAamp循环肿瘤核酸试剂盒提取血浆循环肿瘤细胞DNA（circulating tumor DNA, ctDNA）。分离后的DNA保存在-80 ℃。

### 1.3 统计学方法

采用SPSS 22.0软件进行统计学分析。依据实体瘤疗效评价标准（Response Evaluation Criteria in Solid Tumors, RECIST）1.1标准进行肿瘤治疗疗效评估。治疗后每6-8周进行全身评估，完善血液学检查，评价治疗疗效和病情变化。通用不良事件术语标准（Common Terminology Criteria Adverse Events, CTCAE）5.0评价毒副反应。客观缓解率（objective response rate, ORR）为经过免疫治疗后完全缓解（complete response, CR）和部分缓解（partial response, PR）患者除以总的可评价病例数。疾病控制率（disease control rate, DCR）指肿瘤缩小或稳定且保持一定时间的患者的比例，包含CR、PR和疾病稳定（stable disease, SD）的病例。无进展生存期（progression-free survival, PFS）指有*EGFR*基因敏感突变的肺癌患者从接受免疫治疗开始，到观察到疾病进展（progressive disease, PD）或发生因任何原因死亡的时间。OS为从免疫治疗开始至（因任何原因）死亡的时间。末次随访时间为2020年12月30日。中位随访时间为19.7个月（1.0个月-26.0个月）。*Log-rank*检验进行两组生存比较，ORR和DCR通过*Fisher’s*检验比较，*P* < 0.05为差异有统计学意义。通过*Kaplan-Meier*法分析患者PFS和OS。

## 2 结果

### 2.1 临床病理特征

本研究共纳入患者27例，所有患者均为靶向治疗耐药后接受PD-1单抗联合化疗和贝伐珠单抗治疗，其中免疫治疗包括特瑞普利单抗、信迪利单抗或卡瑞利珠单抗治疗，化疗均为培美曲塞联合铂类方案。4个周期后行PD-1单抗联合维持治疗贝伐珠单抗维持治疗，每3周一次，直至PD或无法耐受不良反应。入组患者中，男12例（44.4%, 12/27），女15例（55.6%, 15/27）。平均年龄（57±10）岁，≤65岁者占63.0%（17/27）。不吸烟或少吸烟者占70.4%（19/27）。按照第八版TNM分期，所有患者入组时均已出现局部或远处转移，所有患者入组前均接受靶向治疗，ECOG评分0分-1分为17例（63.0%），2分为10例（37.0%）。均接受PD-1抑制剂联合化疗和贝伐珠单抗治疗，其中化疗方案均为培美曲塞联合铂类，PD-1抑制剂包括特瑞普利单抗11例（40.8%）、信迪利单抗8例（29.6%）和卡瑞利珠单抗8例（29.6%）（表 1）。

**表 1:** 所有患者的临床病理特征（*n*=27） Clinicopathologic characteristics of all patients (*n*=27)

Characteristics	*n*	%
Age (yr)		
≤65	17	63.0
> 65	10	37.0
Gender		
Male	12	44.4
Female	15	55.6
Smoking		
Yes	8	19.6
No	19	70.4
*EGFR* mutation		
19del	6	22.2
L858R	8	29.6
20Ins	1	3.7
G719X	4	14.9
Combined with T790M	8	29.6
Therapy lines		
The second lines	2	7.4
The third lines	4	14.8
More than the third lines	21	77.8
PD-1 monoclonal antibody		
Toripalimab	11	40.8
Sintilimab	8	29.6
Camrelizumab	8	29.6
PD-1: programmed death 1; EGFR: epidermal growth factor receptor.		

### 2.2 肺腺癌患者肿瘤组织和血液*EGFR*基因突变情况

免疫治疗前所有标本均为*EGFR*基因突变病例，6例*EGFR* 19del突变（22.2%），8例*EGFR* L858R突变（29.6%），合并T790M突变的有8例（29.6%），其他*EGFR*少见突变为5例（18.5%）。其中，1例为*EGFR* 20Ins突变，4例为*EGFR* G719X突变。在8例T790M突变的患者中，血液标本中有2例未发现T790M突变，其余血液标本与组织标本的*EGFR*基因突变完全一致。

### 2.3 疗效分析

通过随访分析，所有患者中，近期疗效达到CR为0例，达到PR为11例（40.7%），达到SD为11例（40.7%），达到PD为5例（18.6%）（表 2）。总体ORR为40.7%（95%CI: 12.5%-66.7%），DCR为81.48%（95%CI: 62.5%-100%）。未合并T790M患者ORR明显优于合并T790M的患者（52.63% *vs* 12.5%, *P*=0.045）（表 2）。所有突变类型的患者中位PFS为6.8个月（95%CI: 0.7-17.1），中位OS为10.2个月（95%CI: 1.0-22.6）。*EGFR* 19del突变、L858R突变、*EGFR*少见突变以及含有T790M突变的4组患者接受免疫四药联合治疗的中位PFS无统计学差异（分别为10.3个月、5.4个月、6.2个月和3.3个月，*χ*^2^=4.15，*P*=0.23），同样，未观察到中位OS的差异（分别为13.1个月、11.5个月、10.4个月和7.3个月，*χ*^2^=2.35，*P*=0.502）（图 1A、图 1B）。而未合并T790M突变的患者PFS较合并T790M突变的患者显著延长（9.2个月*vs* 3.3个月，*χ*^2^=2.81，*P*=0.041），两者OS未见统计学差异（12.2个月*vs* 7.3个月，*χ*^2^=3.22，*P*=0.062）（图 2A、图 2B）。

**表 2:** 不同*EGFR*突变的患者接受免疫联合治疗的近期疗效 Short-term efficacy of combined immunotherapy in patients with different *EGFR* mutations

Response	19del	L858R	Combined with T790M	Other mutation
CR	0	0	0	0
PR	4	5	1	1
SD	2	2	4	3
PD	0	1	3	1
Total	6	8	8	5
ORR	66.67%	62.50%	12.50%	20.00%
DCR	100.00%	87.50%	62.50%	80.00%
CR: complete response; PR: partial response; SD: stable disease; PD: progressive disease; ORR: objective response rate; DCR: disease control rate.				

**图 1:**
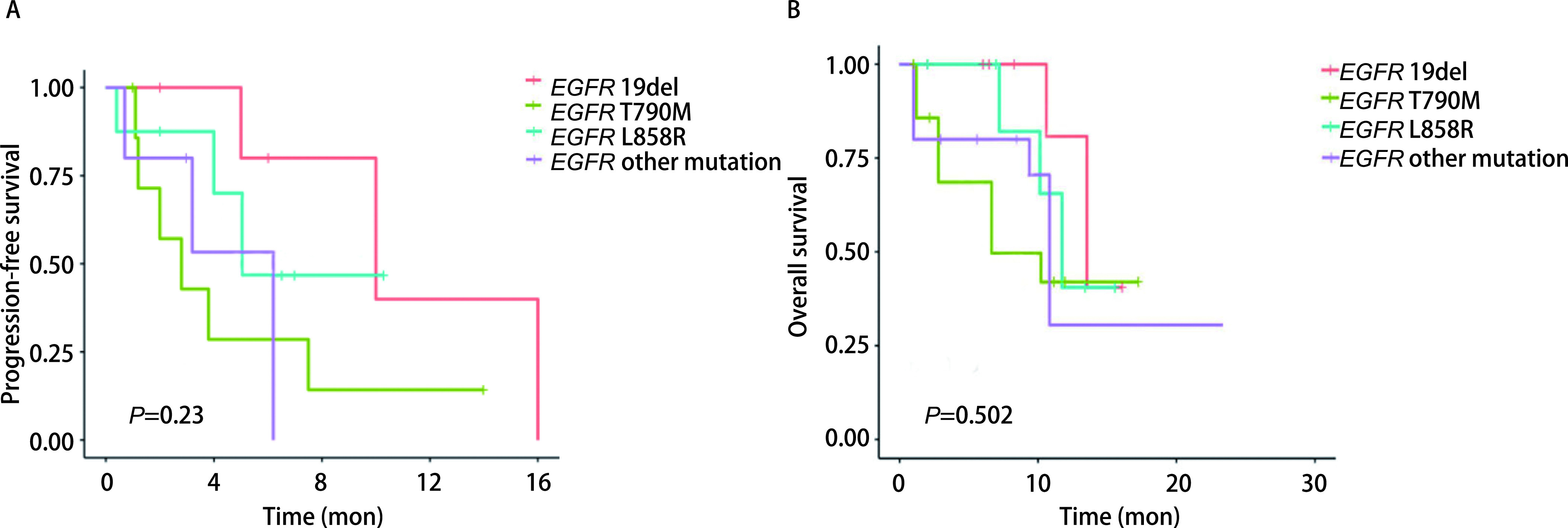
*Kaplan-Meier*法分析免疫联合治疗不同*EGFR*突变患者的无进展生存期（A）和总生存时间（B） *Kaplan-Meier* analysis of progression-free survival (A) and overall survival (B) in *EGFR*-mutant patients treated with combined immunotherapy.

**图 2:**
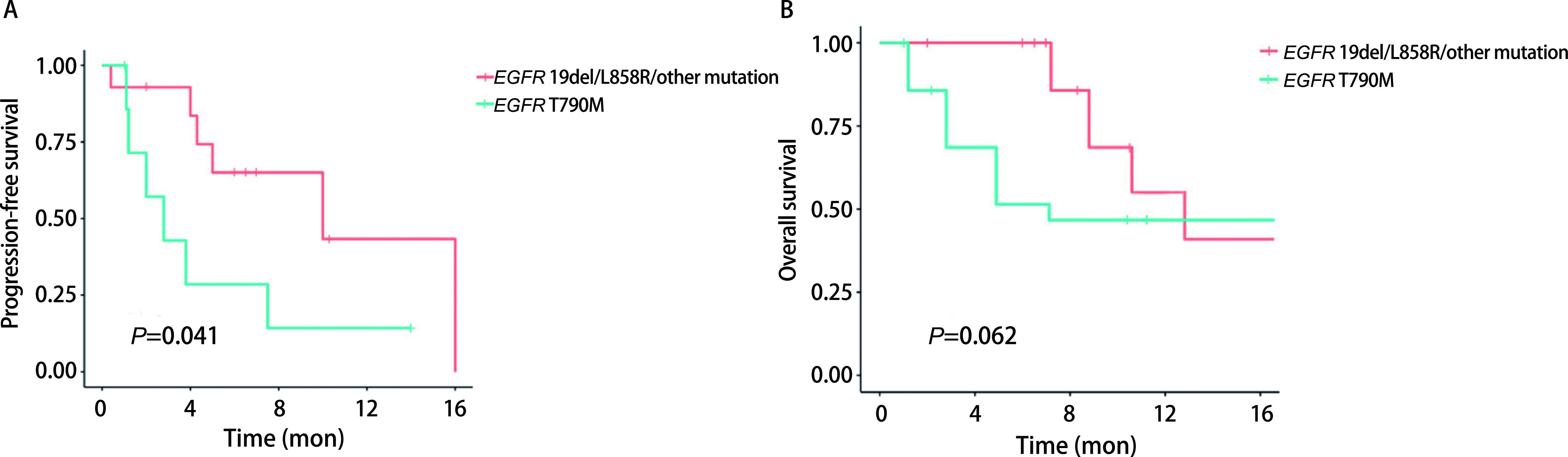
*Kaplan-Meier*法分析免疫联合治疗两个亚组患者的生存情况。A：合并T790M突变与未合并T790M突变的无进展生存期比较；B：合并T790M突变与未合并T790M突变的总生存时间比较。 *Kaplan-Meier* analysis of survival in two subgroups treated with combined immunotherapy. A: Progression-free survival between patients with T790M patients and without T790M mutation; B: Over survival between patients with T790M patients and without T790M mutation.

### 2.4 不良反应

患者发生治疗相关不良反应发生率为92.6%，包括乏力、便秘、谷草转氨酶（aspartate aminotransferase, AST）升高、贫血、白细胞减少、中性粒细胞减少、恶心、皮疹、甲状腺功能减退、肺炎、反应性皮肤毛细血管增生症，未发现新的不良反应（表 3）。其中，≥3级的不良反应为乏力1例，占总体人群的3.7%，停药1个月后可自行缓解。其余均为1级-2级，通过对症支持治疗均可恢复至正常。

**表 3:** 治疗相关不良事件 TRAE

Types of adverse events	All grade	≥Grade 3
Fatigue	10 (37.0%)	1 (3.7%)
Leukopenia	8 (29.6%)	0
Anemia	8 (29.6%)	0
Loss of appetite	8 (39.6%)	0
Nausea	7 (25.9%)	0
Vomit	7 (25.9%)	0
Proteinuria	5 (18.5%)	0
Constipation	4 (14.8%)	0
ALT elevation	4 (14.8%)	0
AST elevation	4 (14.8%)	0
Rash	4 (14.8%)	0
Hypothyroidism	2 (7.4%)	0
Thrombocytopenia	2 (7.4%)	0
Reactive cutaneous capillary hyperplasia	1 (3.7%)	0
All TRAE	25 (92.6%)	1 (3.7%)
TRAE: treatment related adverse event; ALT: alanine aminotransferase; AST: aspartate aminotransferase.		

## 3 讨论

以免疫检查点抑制剂为代表的免疫治疗已成为晚期NSCLC标准治疗方案之一，一线治疗的适应证也已经写入指南。肺癌免疫治疗正向着新辅助治疗方向推进^[11]^。2019年9月，世界肺癌大会（World Conference on Lung Cancer, WCLC）更新了全球Ⅲ期CheckMate 017/057临床研究5年随访生存数据，结果显示，纳武利尤单抗治疗组较化疗组OS延长达5倍（13.4% *vs* 2.6%, HR=0.68, 95%CI: 0.59-0.78）^[12]^。在免疫单药治疗取得突破性进展后，免疫联合放疗、化疗及双免疫治疗使患者的疗效进一步提高，强强联合是肺癌的治疗趋势。但不同的患者疗效仍存在差异，研究者希望从中能筛选出免疫治疗有效的患者进行精准治疗。多数前期研究^[3, 4]^亚组分析均提示*EGFR*/*ALK*基因突变的患者从免疫治疗单药中获益甚微，但有研究^[7, 13, 14]^提示部分*EGFR*/*ALK*基因突变患者仍能从免疫联合治疗中获益，给靶向治疗患者带来新的希望。因此，本研究在真实世界中，评价了靶向治疗耐药后接受免疫联合治疗的疗效，分析免疫治疗在这些患者中的治疗价值。

本研究纳入了接受EGFR-TKI药物治疗耐药的*EGFR*突变患者27例，这些患者后线均接受了PD-1单抗联合化疗和贝伐珠单抗治疗。患者均为晚期肺腺癌，化疗方案为培美曲塞联合铂类化疗。四药联合治疗后，所有突变类型的患者中位PFS为6.8个月（95%CI: 1.8-8.6），中位OS为10.2个月（95%CI: 1.0-16.1）。IMpower150研究亚组分析中，同样探索了*EGFR*突变非鳞癌NSCLC患者一线接受ABCP（化疗联合贝伐珠单抗和阿特利珠单抗治疗组）的中位PFS为9.7个月（95%CI: 6.9-15.2），中位OS为19.0个月（95%CI: 13.5-18.5）^[7]^。本研究与Impower150用药方案相似，四药联合治疗，但应用的是PD-1单抗，同时为后线治疗，患者多线治疗后四药联合的疗效仍能与一线治疗媲美，但OS略差。由此提示，靶向治疗的患者能从后线联合免疫治疗中获益，但尽早地应用免疫联合治疗，可能得到OS的改善。此外，2020年*Lung Cancer*杂志中报道了ATLANTIC研究更新结果，探索了Durvalumab单药三线及后线治疗晚期NSCLC患者的疗效及安全性，其中也包含了驱动基因突变阳性的患者^[13]^。结果显示，携带*EGFR*基因突变的患者，OS为16.1个月，优于*ALK*突变组的6.3个月，但均优于单纯化疗组。进一步印证了本研究的了结论，在后线治疗中免疫治疗仍能发挥重要的作用。

自2018年开启免疫治疗的元年，多项大型国际和国内多中心研究已经开始探索如何将免疫治疗更精准地应用在肺癌治疗中。2020年中国临床肿瘤学会（Chinese Society of Clinical Oncology, CSCO）和最新的2021版美国国立综合癌症网络（National Comprehensive Cancer Network, NCCN）指南均提示对于驱动基因阳性的患者，一线靶向治疗的地位仍不可动摇，尽管PD-L1表达水平较高，但单药免疫治疗仍效果欠佳^[15]^。基于前期的研究，欧盟于2019年3月批准阿特利珠单抗联合贝伐珠单抗以及紫杉醇联合卡铂用于EGFR-TKI耐药后的患者的后线治疗。同时食品药品监督管理局（Food and Drug Administration, FDA）也批准了阿特利珠单抗用于*EGFR*/*ALK*突变的转移性NSCLC患者TKI耐药后的二线治疗^[16]^。此外，国内1.1类创新药物也有了新的临床数据，特瑞普利单抗联合化疗用于EGFR-TKI治疗失败的*EGFR*突变阳性T790M阴性晚期NSCLC患者的Ⅱ期研究，提示意向人群（intention-to-treat, ITT）中总体ORR达到50%，DCR为87.5%^[14]^。与本研究的ORR和DCR相似。抗血管生成治疗和化疗可能对免疫治疗起到协同作用。由此可见，*EGFR*突变的患者靶向治疗耐药后线接受免疫联合治疗仍能获益。基于此，《2020 CSCO非小细胞肺癌诊疗指南》及《2020 CSCO免疫检查点抑制剂临床应用指南》新增注释推荐，特瑞普利单抗联合化疗用于EGFR-TKI治疗失败的*EGFR*突变阳性T790M阴性晚期NSCLC患者。虽然本研究应用了培美曲塞联合铂类的方案，但在四药联合方面，也为*EGFR*突变患者全程管理的后线治疗提供了临床数据。

本项研究的创新之处在于报道了*EGFR*不同突变类型后线接受免疫联合治疗的疗效差异，其中含有T790M突变的患者疗效最差，但L858R与19del以及其他少见突变在远期疗效上未见明显差异。提示这类患者可能需要根据耐药机制探索新的有效治疗方案。由于本研究样本量较少，仍需要更多的数据进一步验证。本研究仍存在一些局限性：①对于肿瘤细胞PD-L1以及肿瘤突变负荷（tumor mutation burden, TMB）未进行统计，但部分研究显示在靶向治疗耐药后对于免疫治疗有效的患者可能PD-L1和TMB明显升高，微环境的重塑打破突变患者原有的免疫豁免机制^[7]^。需要动态研究进一步分析微环境变化在免疫治疗中的作用；②本研究主要阐述了联合免疫治疗在EGFR-TKI耐药后仍能达到预期的治疗获益，但由于不同突变类型的病例较少，亚组分析结论效力不足，需要更大样本的研究探索四药联合的疗效以及耐受性。

总之，驱动基因阳性的患者由于首先接受了靶向治疗，OS较传统治疗已经有了很大的提升。但由于靶向治疗不可避免地出现耐药，耐药后的基因谱复杂，缺乏相应的后续有效治疗策略，探索免疫治疗在这些患者后线治疗的价值对于这部分患者的全程管理至关重要。将来仍需要更有效的分子标志物或联合指标进一步进行精细分层，能更精准地为患者制定有效的治疗方案。
